# A Combined “Hanging Liver Maneuver” and “Intrahepatic Extra-Glissonian Approach” for Anatomical Right Hepatectomy: Technique Standardization, Results, and Correlation With Portal Pedicle Anatomy

**DOI:** 10.3389/fsurg.2021.690408

**Published:** 2021-05-21

**Authors:** Fabio Ferrari Makdissi, Bruno Vinicius Hortences de Mattos, Jaime Arthur Pirola Kruger, Vagner Birk Jeismann, Fabricio Ferreira Coelho, Paulo Herman

**Affiliations:** Digestive Surgery Division, Department of Gastroenterology, Hospital das Clínicas, Instituto do Câncer do Estado de São Paulo (ICESP), University of São Paulo School of Medicine, São Paulo, Brazil

**Keywords:** hepatectomy, colorectal liver metastasis, hanging liver maneuver, intrahepatic glissonian approach, portal pedicle, anatomy

## Abstract

**Background:** The hanging liver maneuver and intrahepatic extra-Glissonian approach are distinct modalities to facilitate safe anatomical liver resections. This study reports a standardized combination of these techniques focusing on safety, results and correlation with portal pedicle anatomy in oncological patients.

**Method:** Combined hanging liver maneuver and intrahepatic extra-Glissonian approach for anatomic right hepatectomy was described stepwise. Portal pedicle anatomy was correlated with the Glissonian approach failure and complications. Clinical characteristics of patients, perioperative outcomes, short and long-term survival rates were analyzed.

**Results:** Thirty colorectal liver metastases patients submitted to the combined approach were evaluated. Anatomical variations of the right portal pedicle were present in 26.6%. Hanging liver maneuver was feasible in 100%, and Glissonian approach in 96.7% despite portal pedicle variations. Mean operative time was 326 min. Mean blood loss was 507 ml. Mean hospital stay was 8 days. There was no 90-day operative mortality and no significant morbidity. Oncological surgical margins were free. Overall and disease-free 5-year survival were 59 and 37%.

**Conclusion:** Regardless of frequent anatomical variations of the right portal pedicle, the hanging liver maneuver, and intrahepatic extra-Glissonian approach can be combined, being useful for anatomical right hepatectomies in a safe and reproducible way in most patients.

## Introduction

Bleeding is one of the main risks during liver resections. Many alternatives of vascular control, parenchymal transection, and division of vascular structures have been reported to decrease intraoperative blood loss and complications of hepatectomies (1, 2). The hanging liver maneuver (3–12) and intrahepatic extra-Glissonian approach (13–26) are two different useful and validated techniques to facilitate safe anatomical liver resections.

The hanging liver maneuver consists of passing a tape or a rubber tube between the anterior surface of the retro-hepatic vena cava and the posterior surface of the liver allowing the liver to be suspended. This tape acts as a guide to a linear anatomic hepatic parenchymal transection. This strategy is also useful to control bleeding and eliminates the need for a wide mobilization of the right liver (3–12).

The intrahepatic extra-Glissonian approach is a strategy for rapid access and control of the main Glissonian pedicles within the liver without the need of dissection of the pedicle elements. This approach is usually fast and offers sectoral control of blood inflow to the liver leading to anatomical ischemic delineation of the area to be resected (13–26). This step is performed before liver parenchyma transection and usually precludes the Pringle maneuver (16, 18, 21–26). A main concern related to this technique during right hepatectomies is the right Glissonian pedicle clamping failure due to incomplete isolation or an extended clamping of the right pedicle with part or all the left pedicle (17, 21, 22, 24–26); this is mostly associated with right portal pedicle anatomic variations (22, 27). The association of the hanging liver maneuver with the Glissonian approach allows the section of the right Glissonian pedicle to be performed at a late stage of right hepatectomies after the complete transection of the hepatic parenchyma. In this way, the right Glissonian pedicle will be completely exposed, facilitating recognition of portal anatomy, and minimizing the risk of damage of the contralateral Glissonian pedicle (15).

Technical standardizations in surgery are critical to increase the safety of surgical procedures. This may be especially important in an academic teaching hospital with a training program for hepato-pancreatic-biliary surgery.

The purpose of this study was to describe in detail a combination of the hanging liver maneuver and intrahepatic extra-Glissonian approach for anatomical right hepatectomy in patients with colorectal liver metastases. The preoperative portal pedicle anatomy was assessed by computed tomography (CT) or magnetic resonance imaging (MRI), and correlated to surgical outcomes. Herein we present our experience with this standardized combined approach in an academic teaching tertiary hospital.

## Methods

The combination of hanging liver maneuver and the intrahepatic extra-Glissonian approach for anatomical right hepatectomy has been standardized and used for the treatment of oncological patients at our institution, a tertiary teaching hospital with a training program in hepatobiliary surgery.

Patients submitted to anatomical right hepatectomy using the combination approach for the treatment of colorectal liver metastases were enrolled in our prospective database and reviewed. Clinical and surgical characteristics of the patients, early outcomes, as well as long-term results were analyzed.

The inclusion criteria were: (a) multiple metastases (>3) with deep location within the right liver (>2 cm in depth); (b) large tumors (>5 cm) in the central area of the right liver; (c) minimum of 6 months' follow-up after surgery; and (d) liver remnant volume of at least 30%.

Exclusion criteria were: (a) tumor invasion of the main right Glissonian pedicle; (b) need for major or anatomical resections in the left liver; (c) tumor infiltration to the retro-hepatic avascular plane or Inferior Vena Cava; (d) liver cirrhosis; (e) previous hepatic hilum surgical manipulation.

All cases were previously discussed in multidisciplinary meeting including liver surgeons, radiologists, and clinical oncologists.

### Preoperative Evaluation of Portal Anatomy

**Figure 1:**
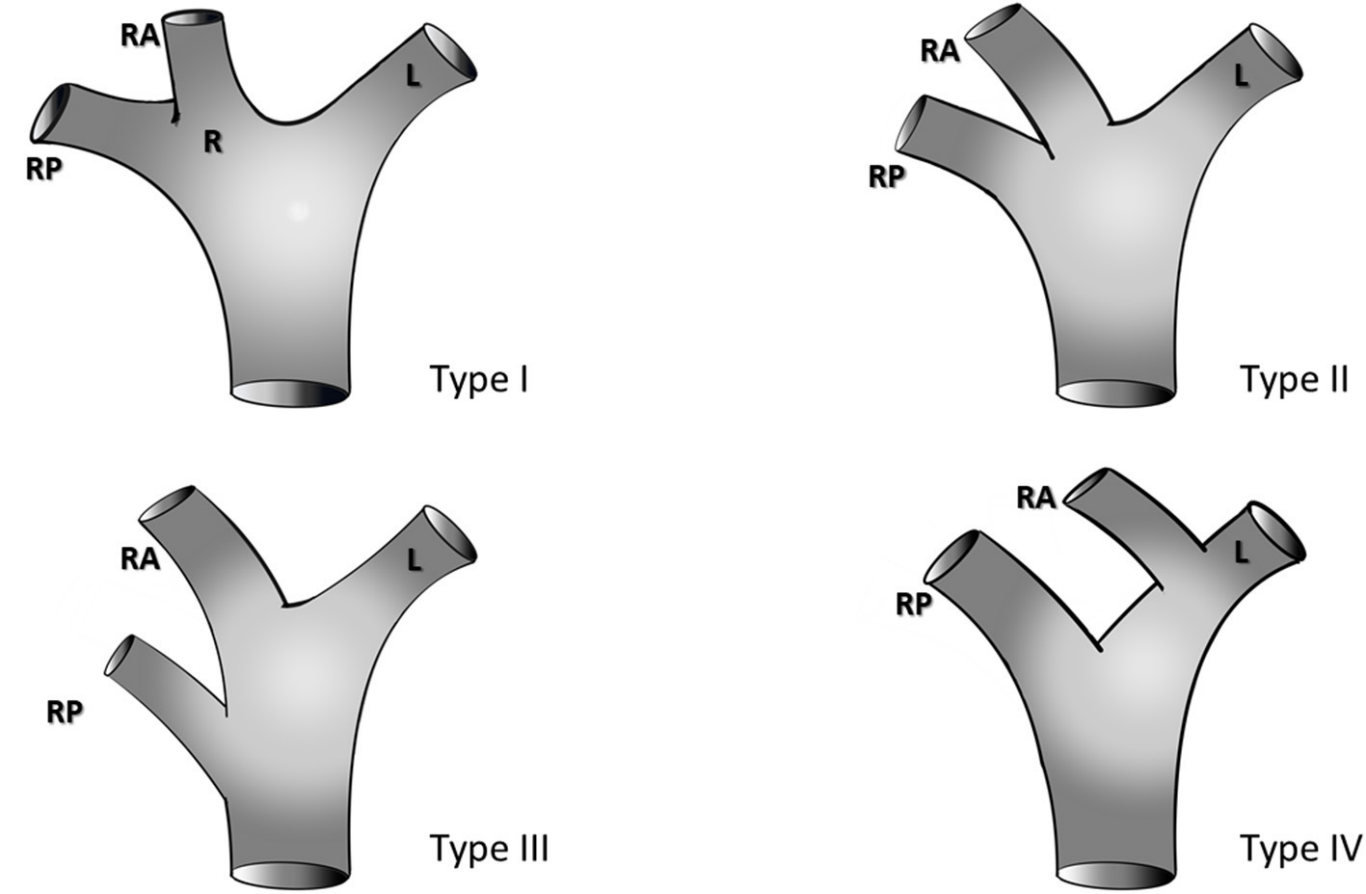
Portal vein anatomy according to the classification of Cheng et al. (28), R, Right portal pedicle; RA, right anterior portal pedicle; RP, right posterior portal pedicle; L, left portal pedicle.

Preoperative triphasic contrast-enhanced CT scans or MRI was performed. The portal pedicle anatomy was evaluated and classified according to Cheng et al. (28) classification (Figure 1):

- Type I (classical anatomy) - division of the portal pedicle into right and left branches immediately before reaching the liver with further division of the right portal branch into anterior and posterior sectorial branches;
- Type II - portal trifurcation in which the left portal pedicle, right anterior, and right posterior pedicles share the same origin;
- Type III - the right posterior sectoral pedicle comes directly from the main portal pedicle independently of the right anterior portal pedicle; it sometimes arises first at the lower part of the hepatic hilum;
- Type IV - the right anterior sectoral pedicle comes from the left portal branch;
- Type V - other variations.

### Surgical Technique

The patient is positioned in a supine position. A supra-umbilical midline incision with right subcostal extension is then performed (inverted L-shaped laparotomy), and a retractor is placed. Cholecystectomy is performed if the gallbladder is in place. The round and falciform ligaments are divided until the right coronary ligament.

Intraoperative ultrasound is then performed to assess the presence of any left hepatic lobe lesions, to define the surgical margin, and to identify the middle hepatic vein that should be preserved.

#### Hanging Liver Maneuver

Upper and lower tunnels were created in the retro-hepatic space between the liver and the vena cava following previously reported precepts for the hanging liver maneuver (3, 4, 6, 8, 9):

- Upper dissection at the supra-hepatic vena cava: The falciform ligament division is extended superiorly, and the cranial right coronary ligament is opened to expose the anterior aspect of the cava vein between the right hepatic vein (RHV) and the middle hepatic vein (MHV). The space between RHV and MHV is dissected downwards (cranio-caudal direction) along with the vena cava axis. This dissection must be performed carefully with a right-angle clamp through the soft tissue between the vena cava adventitia and Laennec's capsule for an extension of about 3–4 cm. Any kind of resistance during dissection should be interpreted as a potential accessory vein or misdirection of the clamp to the vena cava wall or to the liver capsule. This tunnel is usually anterior and somewhat to the right side of the vena cava.
- Inferior dissection at the infra-hepatic vena cava: A small opening of the peritoneum is created at the intersection between the anterior aspect of the infra-hepatic vena cava and the caudate lobe giving access to the virtual space between the vena cava adventitia and the liver capsule (Laennec's capsule). A long blunt-tip clamp is gently inserted in this space (11 o'clock position of the vena cava) heading cranially to the previously dissected supra-hepatic area for the creation of a tunnel between the liver and retro-hepatic vena cava. The identification of the retro-hepatic forceps can be performed by ultrasound as well as the eventual presence of accessory veins.

**Figure 2:**
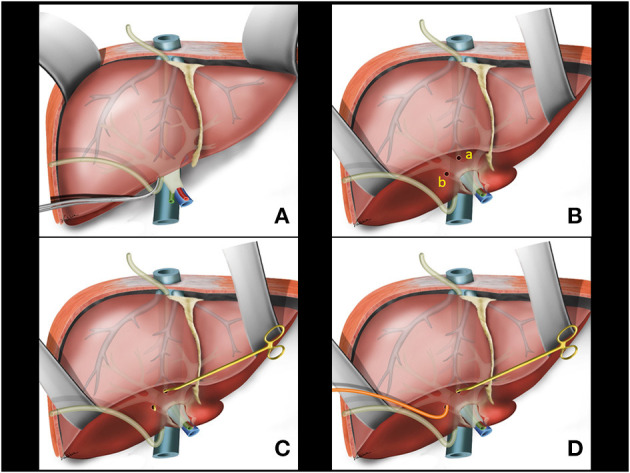
**(A)** Hanging Liver Maneuver: naso-gastric tube passage in the retrohepatic space. **(B)** Small openings in the liver capsule at the base of segment 4B (a) and between the right posterior sector and the caudate lobe (b). **(C)** Glissonian pedicle retriever inserted deep into the liver parenchyma with a rotating movement between previous liver capsule openings. **(D)** A rubber tube is attached to the instrument tip.

Communication between the upper and lower tunnels can be achieved from bottom to top or from top to bottom with the aid of digital tactile maneuvering or a naso-gastric tube passage (Figure 2A).

#### Intrahepatic Extra-Glissonian Approach of Right Pedicle

**Figure 3:**
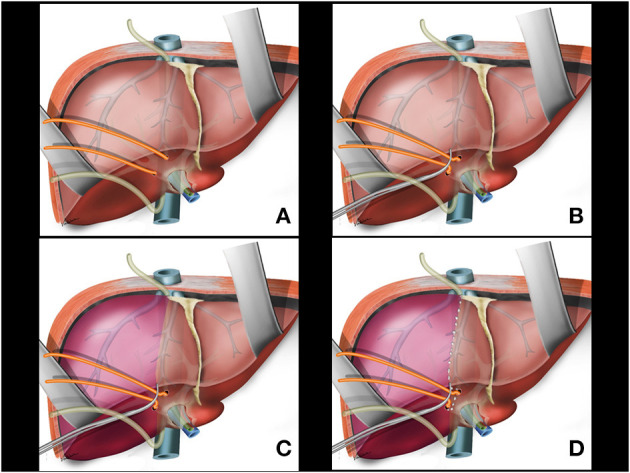
**(A)** Glissonian pedicle retriever is withdrawn and the right glissonian pedicle is isolated by the rubber tube. **(B)** The rubber tube is pulled for occlusion of the isolated pedicle. **(C)** Ischemic delimitation of the right hepatic parenchyma is observed. **(D)** Ischemic transition line is demarcated with electrocautery.

Following the previously reported precepts for intrahepatic extra-Glissonian approach of the right pedicle (16), a small opening is made in the liver capsule at the base of segment 4B just above the hilar plate and slightly to the right. Another small opening is made in the liver capsule between the right posterior sector and the caudate lobe (segment 9) (Figure 2B). An atraumatic Glissonian pedicle retriever (reported elsewhere) (29) or a large right angled clamp (Mixter or Gray clamps) is softly inserted deep into the liver parenchyma with a rotating movement between these incisions and encircling the right pedicle (Figure 2C). A rubber tube is then attached to the instrument tip (Figure 2D), and the retriever is withdrawn (Figure 3A). The rubber tube is then pulled for the occlusion of the isolated pedicle in a “tourniquet maneuver” (Figure 3B). Ischemic delimitation of the right hepatic hemi liver is then observed to assure that the right Glissonian pedicle was included within the rubber tube (Figures 3C,D). At this point, intraoperative Doppler ultrasound can be performed to confirm that the entire pedicle is encircled by the rubber tube.

#### Combination of the Two Techniques (Hanging Liver Maneuver and Intrahepatic Extra-Glissonian Approach) of Right Pedicle

**Figure 4:**
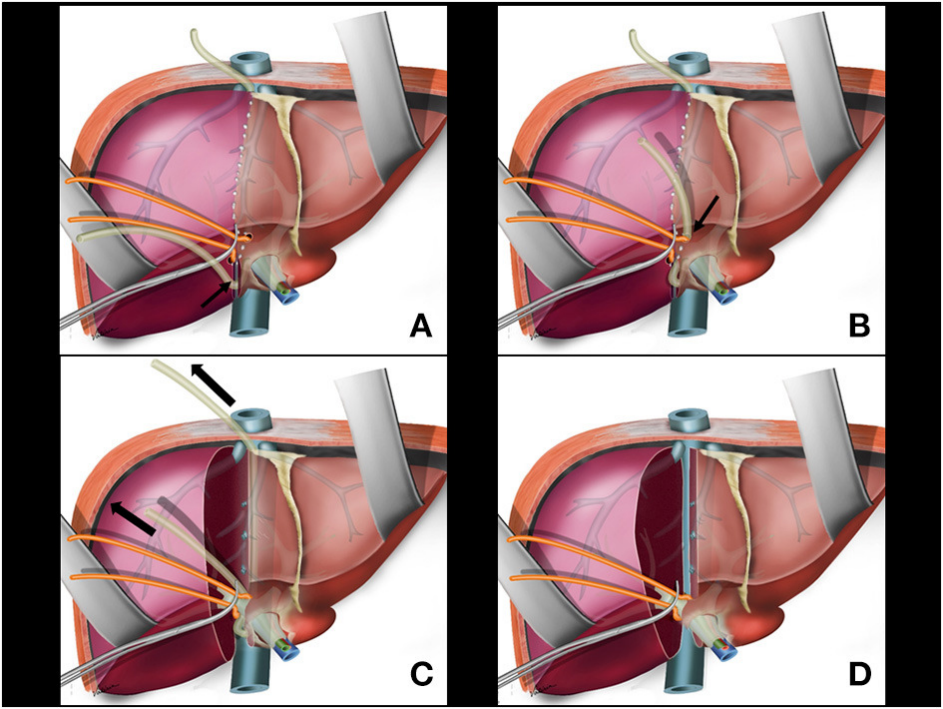
**(A)** Partial transection of liver parenchyma between the caudate lobe and the right posterior sector; the nasogastric tube of the liver hanging manouver is fitted in this space (arrow). **(B)** The nasogastric tube used in the hanging maneuver is brought to the anterior aspect of the right glissonian pedicle (arrow). **(C)** During parenchymal transection, upwards traction is applied on the nosogastric tube (arrows). **(D)** Main right pedicle without liver tissue around it. Anterior aspect of retrohepatic vena cava is exposed.

A partial transection of liver parenchyma between the caudate lobe and the right posterior sector is performed with cautery and bipolar energy communicating with the previous opening for Glissonian pedicle isolation. This opening allows for the nasogastric tube of the liver (from the hanging maneuver) to be fitted (Figure 4A).

A right angled clamp is passed again through the space created for isolation of the right Glissonian pedicle. This maneuver can be facilitated with traction of the rubber tube encircling the Glissonian pedicle. The nasogastric tube used in the hanging maneuver is brought to the anterior aspect of the right Glissonian pedicle to protect the pedicle during transection (Figure 4B). At this time, the nasogastric tube would involve only the hepatic parenchyma and the main venous branches draining segments 5 and 8 in the middle fissure, with no pedicle structures included.

#### Liver Transection

Glisson's capsule is marked with cautery preferably 0.5–1.0 cm within the ischemic line. The marked area is checked by ultrasound to ascertain the oncological margin, identify the middle hepatic vein, and recognize its main branches. Hepatic transection is initiated from the antero-inferior border of the liver (transition between segments 4B and 5) heading the tractioned nasogastric tube direction as a guide. For liver transection, CUSA (Cavitron Ultrasonic Surgical Aspirator; ValleyLab, Boulder, Colorado, USA) and/or bipolar energy associated with ligature and section of major hepatic veins (V5 and V8) is performed.

During parenchymal transection, upwards traction is applied by the second assistant on the nasogastric tube by holding one of the ends of the tube and keeping the other end attached to the retractor. The nasogastric tube elevates the liver making it easier to be transected while constantly guiding the surgeon toward the correct plane leading to a vertical transection line along the shortest route. The traction on the nasogastric tube can also be regulated to provide control in instances of venous bleeding helping to identify the bleeding vessel (Figure 4C).

Lifting the nasogastric tube up and keeping the hanging maneuver opens the transection plane allowing identification, as well as preservation, of the middle hepatic vein as well as protection of the IVC. This strategy also provides an “open-book” effect on both lobes of the liver as the dissection progresses. The enhanced exposure can contribute to better hemostasis in the transected surface.

#### Right Pedicle(S) and Right Hepatic Vein(S) Transection

The right Glissonian pedicle will be exposed at the end of liver transection. It is only surrounded by the rubber tube and is ready to be securely sectioned. This technique allows the complete exposition of the main right pedicle. At this time, counter traction on the rubber tube to the left side is applied for application of a linear stapler (Figure 4D), ensuring that the confluence of the bile ducts is not accidentally ligated (26). To ensure that the contralateral bile duct is preserved and has not been inadvertently occluded, cholangiography can be performed before the section of the right glissonian pedicle. When the portal pedicle variation Type II or III is present, the right anterior and posterior pedicles are independently exposed after parenchyma transection, and sectioned apart.

**Figure 5:**
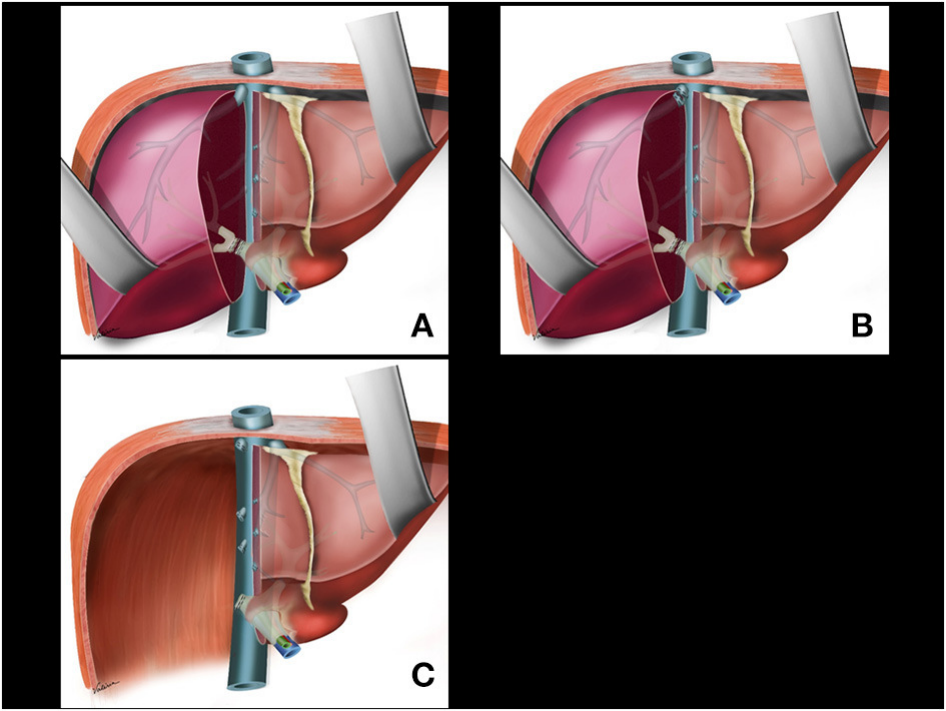
**(A)** Division of the right pedicle. **(B)** Right hepatic vein and right accessory veins transection. **(C)** Last step of the surgery is to release the liver from the right aspect of cava vein, right ligaments, adrenal gland, and diaphragm including tumor adhesions if present.

There is no blood inflow to the right liver after separation of the right pedicle (Figure 5A) or right anterior and posterior pedicles. The remaining major vessels will be the right hepatic vein and the right accessory veins. The anterior surface of the retro-hepatic vena cava is completely exposed at this time making easier to dissect the remaining right veins from medial to right lateral. Another possibility is transection of the right vein with a stapler placed in parallel to the right lateral aspect of vena cava (Figure 5B). The last step of the surgery releases the liver from the right ligaments from medial to right lateral and from caudal to cranial planes. If tumor adhesions with the diaphragm, Gerot's capsule, right adrenal, and even on the right side of the retro hepatic vena cava is present, the oncological resection is performed at this time. The remnant left liver is fixed in its orthotopic position (Figure 5C); an abdominal drain is not routinely used.

### Variables

The feasibility of the procedure was assessed by success in performing the hanging liver maneuver and intrahepatic extra-Glissonian approach. The rate of patients with normal anatomy and anatomical variations of portal pedicle—as well the type of anatomical variations—were correlated to failure in achieving the intrahepatic Glissonian approach for the right pedicle (rate of incomplete clamping of the right Glissonian pedicle or extended clamping to the left side). Section of the right portal pedicle in a single trunk or right anterior and posterior independently was also evaluated.

The safety of the procedure was assessed as occurrence of complications such as bleeding, blood transfusion, liver failure, iatrogenic injuries to the left pedicle, biliary fistula, and length of hospitalization. The 90-day mortality was also assessed. The Clavien–Dindo classification was used to further account for the severity of complications. Secondary outcomes were oncological results such as oncological margins, overall survival, and disease-free survival.

### Follow-Up

The follow-up protocol included contrast-enhanced computed tomography or magnetic resonance every 3 or 4 months for the first 2 years and every 6 months thereafter.

## Results

From 2014 to 2020 there were 725 liver resections at our institution, being 104 right hepatectomies (85 for neoplasic and 19 for benign desease). Right hepatectomy was used for malignant diseases treatment in: 47 colorectal liver metastases, 21 hepatocellular carcinomas, 4 intrahepatic cholangiocarcinomas, 4 hilar cholangiocarcinomas, 2 neuroendocrine tumor liver metastases, 7 non-colorectal, non-neuroendocrine liver metastases.

Applying the inclusion and exclusion criteria, thirty patients with colorectal liver metastases (22 males, 8 females), with a mean age of 59.6 ± 14.9 years were submitted to the combined approach for right hepatectomies. All surgeries were performed by fellows in a hepatobiliary surgery training program under the assistance and supervision of a senior surgeon.

The hanging liver maneuver was feasible in all patients (100%). The Glissonian pedicle retriever was used in all cases without inadvertent Glissonian pedicle injury or significant bleeding. The intrahepatic extra-Glissonian approach for the right pedicle was feasible in 29/30 patients (96.7%).

The classical anatomy of the portal pedicle (Type I) was present in 22 patients (73.3%), and anatomical variations of the portal anatomy included Type II: 4 (13.3%), Type III: 3 (10%), and Type IV: 1 (3.3%). There were no Type V anatomical variations. The intrahepatic extra-Glissonian approach failed in the only patient with Type IV anatomy. In this patient, the technique could only include the posterior right sector pedicle as confirmed by Doppler ultrasound. The anterior pedicle was identified after liver transection.

When classical anatomy (Type I) was present, the right Glissonian pedicle was stapled in its common trunk; and when anatomical variation Types II or III was present, the anterior and posterior glissonian pedicles were isolated and stapled independently.

There were no intraoperative complications related to the hanging maneuver and no injury of the left portal pedicle related to the Glissonian approach.

Mean operative time was 326 ± 92 min. Associated wedge liver resections were performed on the left liver in 14 patients (a total of 27 wedge resections with a range of 1–7 lesions). The mean estimated blood loss was 507 ± 388 ml, and three patients required perioperative blood transfusion (transfusion rate of 10%). An intermittent Pringle maneuver was used in one patient because of failure in achieving the anterior pedicle isolation through the intrahepatic Glissonian approach (Type IV patient).

Outcomes were uneventful with no significant morbidity; all patients presented Dindo-Clavien complications score lower than three. No clinical or laboratory signs of liver failure were observed. The mean hospital stay was 8.0 days, and there was no 90-day mortality. The oncological surgical margins were free in all patients (R0 resections). The operative data and postoperative complications are summarized in Table 1.

**Table 1:** Operative variables and postoperative complications.

**Variable**	
Operative time, mean–SD, in min	326 ± 92
Estimated blood loss, mean–SsD, ml	507 ± 388
Blood transfusion during hospitalization, *n* (%)	3 (10)
ICU stay, mean–SD, days	2.0 ± 1.5
Hospital stay, mean–SD, days	8.0 ± 3.8
Wound infections, *n* (%)	0 (0)
Bile leak, *n* (%)	0 (0)
Intra-abdominal abscess, *n* (%)	0 (0)
Postoperative bleeding, *n* (%)	0 (0)
Reoperation, *n* (%)	0 (0)
Post-operative liver failure, *n* (%)	0 (0)
Complications (Dindo–Clavien ≥ 3), *n* (%)	0 (0)
Liver failure (Child-Pugh >7)	0 (0)
90 days mortality	0 (0)
R0 resection, *n* (%)	30 (100)

*SD, standard deviation; ICU, intensive care unit*.

After a median follow-up of 39 months (range 8–79), recurrence was seen in 17 patients (56%); 4 (4/17) had liver recurrence only, and 13 (13/17) had extrahepatic or hepatic and extrahepatic recurrence. Of the patients with exclusive liver recurrence, one was submitted to re-hepatectomy and one to percutaneous radiofrequency ablation; both are currently free of disease. Eight patients died due to oncological disease progression. The overall and disease-free 5-year survival rates were 59 and 37%, respectively.

## Discussion

The aim of this study was to standardize the combined technique and evaluate the results of this strategy using the hanging liver maneuver and intrahepatic extra-Glissonian approach to perform anatomical right hepatectomies for oncological patients in a tertiary teaching hospital.

Whenever possible, our group's policy favors liver-sparing resections while always respecting the oncological principles. Patients included in this study had indications for right hepatectomy by the number and/or size and/or location of the neoplastic disease.

Anatomical right hepatectomies are usually preceded by a wide mobilization of the right lobe of the liver. To avoid extensive mobilization and rotation of the right liver to the left side, the hanging liver maneuver was proposed in 2001 by Belghiti et al. (3). Subsequent publications offered more details and practical aspects of the technique as well as association with other maneuvers for liver resection. The results underscore the safety and effectiveness of the hanging liver approach while validating the technique (4–12). A key concept of this technique is to release the right liver as a last step of the surgery after complete transection of the hepatic parenchyma without an increased risk of retro-hepatic vena cava injury caused by a “blind” bipartition. The hanging liver technique is particularly important in the presence of massive lesions on the right liver and/or tumor adhesions with the diaphragm, Gerot's capsule, right adrenal, and even on the right side of the retro hepatic vena cava. Additional advantages of this maneuver include: (a) decreased ischemic effects on the left remnant liver due to “rotation” of the liver from right to left leading to “torsion” of the Glissonian pedicles and hepatic veins; (b) decreased systemic hemodynamic effects due to impairment of liver and inferior vena cava blood return; (c) decreased risk of tumor cells spreading due to the manipulation of the tumor; and (d) decreased risk of tumor rupture (oncological violation) and exposure with the potential for bleeding (3–12).

Another key point during hepatectomies is blood inflow control in order to reduce the threats of substantial bleeding. Many techniques can regulate blood inflow to the liver and the selective Glissonian pedicle isolation (intrahepatic extra-facial or extrahepatic extra-fascial) are useful and validated approachs.

The extrahepatic extra-fascial approach, by detaching the liver parenchyma from the main Glissonian pedicles at the hepatic hilum, was introduced by Couinaud and Takasaki et al. (30–32). The intra hepatic extra-fascial (or extra-Glissonian) approach to control the right Glissonian pedicle inflow before liver transection was first described by Galperin and Karagiulian (13) and later refined and disseminated by Launois and Jamieson (14, 15) as well as by Machado et al. (16, 18, 19). These techniques are based on the intrahepatic retrieval of the Glissonian pedicles, which can be encircled in a less traumatic way using a right-angled dissector or a Gray clamp. The basis and anatomical landmarks are described elsewhere (33) along with the standardized technique for intrahepatic Glissonian pedicle access to right and left liver segments for anatomical hepatectomies (16, 18, 19, 34). An instrument for liver pedicle retrieval was later designed allowing an atraumatic intrahepatic extra-Glissonian pedicle isolation in a soft and gentle maneuver preventing rupture of intrahepatic structures as Glissonian pedicle structures and hepatic veins (29). The advantages of this technique are the straightforward access and control of main Glissonian pedicle without time-consuming dissection of the Glissonian elements. Once isolated, the Glissonian pedicle can be clamped leading to a precise ischemic delineation for anatomical liver resection before liver transection. This ischemic pre-transection condition may preclude total clamping blood inflow control (Pringle maneuver) with maintenance of perfusion to the future liver remnant. This reduces the potential ischemic damage to the remnant liver resulting in better liver function after liver resection.

A combination of the hanging liver maneuver and the intrahepatic extra-Glissonian approach was theorized in the textbook “The Posterior Intrahepatic Approach in Liver Surgery” (Launois and Jamieson, (15)), and was applied in 30 patients who underwent right hepatectomy in our institution.

The main criticisms to the intrahepatic extra-Glissonian approach are the fear of inadvertent pedicle injury or hepatic vein damage during insertion of the retriever for pedicle isolation; and concerns related to incomplete clamping or even extended contralateral pedicle clamping leading to the injury of the left bile duct after right pedicle ligation or stapling (17, 21, 22, 24, 25). In this study, no inadvertent pedicle injury or massive bleeding was observed with the use of the Glissonian pedicle retriever (29).

Previous studies reported 69–100% feasibility for the Glissonian approach in major hepatectomias (16–19, 21–25). Most of the failures were associated with portal pedicle anatomic variations (22). A recent anatomical study based on multiphasic CT and routine 3D reconstruction characterized the anatomical variations of the right Glissonian pedicle at risk of clamping failure (27). The authors studied 346 patients and found that the classical anatomy was present in 245 patients (71%). There was a risk of right Glissonian pedicle clamping failure (33.8%) related to anatomical variations of the portal pedicle or an angle of <50° between the portal vein and the left portal vein. The risk of clamping accidentally the left pedicle was 16% and of incomplete clamping was 17.8% (27).

In our study, results were similar with data highlighted in other studies showing that anatomical variations of the right portal pedicle are frequent (22, 27, 35–37), and it was present in 26.6% of the cases. However, we found a high rate of success in applying the Glissonian approach technique (96.7%) with a low rate of complications. The reason for our good results may be our policy to leave the section of the right Glissonian pedicle as a final step of the surgery after the complete transection of the hepatic parenchyma allowing the exposure of the entire right Glissonian pedicle before ligation or stapling.

The majority of cases present a classical anatomy (Cheng's type I), where the right portal pedicle is a common trunk before the segmentation of the anterior and posterior right pedicles. These can be safely stapled, mainly when counter-traction on the other side (left side) is applied to reduce the risk of contralateral pedicle injury (26). In anatomical variations types II and III, the anterior and posterior right branches are independently exposed after transection of the hepatic parenchyma being sectioned apart. Previous studies have shown frequent anatomical variations in first-order Glissonian pedicle branches, mostly dissociation between vascular and biliary structures, but these anatomical variations and dissociations rarely occur in second-order Glissonian branches (28, 35). We had no complications related to extended clamping, biliary or vascular lesions of the left portal pedicle. Incomplete clamping of the right pedicle occurred in only one patient with type IV anatomical variation (right anterior pedicle arising from the left portal branch). In this situation, only the right posterior pedicle can be isolated, and the right anterior pedicle is approached by the anterior access. The types IV and V anatomical variations hinder the technique described herein, but fortunately, these variations are less frequent and can be easily recognized in preoperatively planning imaging scans (38, 39), allowing a different approach to be planned.

Regardless of the technique applied (intra-fascial, extrahepatic extra-fascial, and intrahepatic extra-fascial), anatomical variations should always be considered in liver resections, and caution should be taken in patients with aberrant anatomy. Thus, we understand that a combination of the hanging liver maneuver and the intrahepatic Glissonian approach can be safely applied for right hepatectomies when the classic portal anatomy (type I) or types II and III anatomical variations are present. In types II and III, a separate and independent section of the right anterior and posterior pedicles is strongly recommended; in our standardization, these pedicles are individually exposed and easily recognizable allowing a safe section. The combined approach should be avoided in the rare cases of anatomical variations types IV and V.

The technique described herein is feasible and reproducible in most cases with good surgical and oncological results. We recognize that this technique allows fellows in hepatobiliary surgery, supervised by a senior surgeon, to perform a complex procedure in a fast and standardized way. This procedure allowed a combination of the advantages of two standardized and validated techniques in order to perform a safe and straightforward right hepatectomy.

## Conclusions

The hanging liver maneuver and intrahepatic extra-Glissonian approach can be combined to achieve anatomical right hepatectomies in a safe and reproductive way. Preoperative anatomical evaluation of portal pedicles is essential for the success of this technique. Regardless of frequent anatomical variations, this standardized combination of techniques might be an optimal approach for anatomical right hepatectomy in most patients treated for colorectal liver metastases.

## Data Availability Statement

The original contributions presented in the study are included in the article/supplementary material, further inquiries can be directed to the corresponding author/s.

## Author Contributions

All authors have participated in conception and design, or analysis and interpretation of the data, drafting the article or revising it, and approving the final version.

## Conflict of Interest

The authors declare that the research was conducted in the absence of any commercial or financial relationships that could be construed as a potential conflict of interest.
